# How sensitive are the evaluations of a school’s effectiveness to the selection of covariates in the applied value-added model?

**DOI:** 10.1007/s11092-022-09386-y

**Published:** 2022-05-23

**Authors:** Jessica Levy, Martin Brunner, Ulrich Keller, Antoine Fischbach

**Affiliations:** 1 Luxembourg Centre for Educational Testing, Faculty of Humanities, Education and Social Sciences, University of Luxembourg, 11, Porte des Sciences, L-4366 Esch-sur-Alzette, Luxembourg; 2 Department of Education, University of Potsdam, Karl-Liebknecht-Str. 24-25, 14476 Potsdam, Germany

**Keywords:** Value-added models, Elementary school, Accountability, Multilevel models

## Abstract

There is no final consensus regarding which covariates should be used (in addition to prior achievement) when estimating value-added (VA) scores to evaluate a school’s effectiveness. Therefore, we examined the sensitivity of evaluations of schools’ effectiveness in math and language achievement to covariate selection in the applied VA model. Four covariate sets were systematically combined, including prior achievement from the same or different domain, sociodemographic and sociocultural background characteristics, and domain-specific achievement motivation. School VA scores were estimated using longitudinal data from the Luxembourg School Monitoring Programme with some 3600 students attending 153 primary schools in Grades 1 and 3. VA scores varied considerably, despite high correlations between VA scores based on the different sets of covariates (.66 < *r* < 1.00). The explained variance and consistency of school VA scores substantially improved when including prior math and prior language achievement in VA models for math and prior language achievement with sociodemographic and sociocultural background characteristics in VA models for language. These findings suggest that prior achievement in the same subject, the most commonly used covariate to date, may be insufficient to control for between-school differences in student intake when estimating school VA scores. We thus recommend using VA models with caution and applying VA scores for informative purposes rather than as a mean to base accountability decisions upon.

**Supplementary Information:**

The online version contains supplementary material available at 10.1007/s11092-022-09386-y.

How can we find out how schools influence the development of students’ achievement? Drawing on statistical methods in agriculture agricultural analyses (e.g., Kupermintz, 2003) value-added (VA) models have been developed as a statistical tool to estimate educational effectiveness at different levels. An early reference to VA models can be found in Hanushek (1971) and one of the first educational VA model was the “Tennessee Value-Added Assessment System” (Sanders & Horn, 1994). Since then, various targets for VA models have been identified, involving teachers and schools, or less frequently also school principals. Common to all VA models is their aim to “make fair comparisons of the academic progress of pupils in different settings” (Tymms, 1999, p. 27). In particular, VA models targeting schools (to which we refer as school VA models) are applied to find the “value” (i.e., the school VA score) that was added by schools to students’ achievement, independent of students’ backgrounds (e.g., Amrein-Beardsley et al., 2013). Conceptually, this means that the actual achievement attained by students attending a certain school is juxtaposed with the achievement that is expected for students with the same starting characteristics (e.g., pretest scores). A positive effect of attending a certain school is suggested when actual achievement is better than expected achievement (i.e., a positive VA score is observed).

VA models are often used for accountability and high-stakes decisions, not only about teachers, but also to allocate financial or personal resources to schools. In other words, the use of VA models is often highly consequential. It is thus a highly political topic, especially in the USA, where many states have implemented VA-based evaluation systems, particularly on the teacher level (Amrein-Beardsley & Holloway, 2017; Kurtz, 2018) even though the consequential use of VA models seems to be decreasing again in many states (Close et al., 2018). Importantly, the application of VA models for high-stakes decisions at the school level is not restricted to the USA. Other countries, such as France or the UK, are also using school VA models for accountability purposes (e.g.; Duclos & Murat, 2014; Perry, 2016).

Given the far-reaching impact of VA scores, it is surprising that there is no final consensus on how to obtain the best estimate of school VA scores (Levy et al., 2019; Everson, 2017; but see Koedel et al., 2015 for a review on the consensus for the calculation of teacher VA). This lack of consensus can be observed for various aspects of the school VA model, including the applied statistical model, methodological adjustments, and the selection of covariates to compute the VA score. In particular, school VA scores are often interpreted as causal effects of (in)effective pedagogical practices within a certain school (for a critical discussion on causal inferences based on school VA scores, see, Reardon & Raudenbush, 2009). Conceptually speaking, as school VA scores represent the part of the variance in performance measures that cannot be explained by the covariates in the model, the question of which covariates should be included in the VA model is of utmost importance. Whereas there seems to be consensus that prior achievement is the primary choice for a covariate (e.g., Levy et al., 2019; Casillas et al., 2012; Hanushek, 1971; Ray, 2006), it is still not clear whether it should be the only covariate, or whether (and if so, which) other covariates could contribute to school VA scores to best approximate a school’s effectiveness without any bias. In the context of VA scores, “unbiased” means that differences in school VA scores between schools are—to the greatest possible extent—due to true differences in school effectiveness rather than to between-school differences in student intake, thus that the forecast is as unbiased as possible. For example, if a fictive School A selected only the most motivated students, the effectiveness of this school would be overrated if the school VA scores were only estimated with prior achievement as a predictor without considering students’ motivation. This would lead to wrong information on school report cards, which might lead to parents wrongfully wanting to send their children to this school. In high-stakes contexts, this might even mean that School A receives more resources than other schools. This example shows that the choice of covariates can have an immediate consequence on school development. The overarching goal of the present study is therefore to systematically analyze the extent to which school VA scores for mathematics and language achievement are sensitive to the selection of covariates in the applied VA models and which practical implications covariate selection can have for individual schools. This allows to investigate whether the current practices of school VA models can lead to biased estimates of school VA scores and in how far adding additional covariates can help reduce this bias in school VA scores.

## *Practical issues to obtain unbiased school VA scores*

There is a broad literature on sources of bias when estimating school VA scores (see, e.g., Harris & Anderson, 2013; van de Grift, 2009; and see the Standards for Educational & Psychological Testing, 2014, for a general discussion in psychometrics). Importantly, VA models contain three parts: (a) the outcome, (b) the covariates that are used to predict the outcome, and (c) the residual term that depicts the school VA score. There is broad consensus that valid school VA scores can only be obtained when using valid and reliable outcome measures, such as standardized tests from educational large-scale assessment programs (Braun, 2015). However, there is some debate on the selection of covariates, as will be described in the following.

## Covariate selection for school VA models

Covariate selection (i.e., the choice of “correct” covariates) is important in the estimation of causal effects in observational studies (Steiner et al., 2010). As the aim of covariate selection is to reduce bias in causal estimates, its importance is also evident for the estimation of school VA scores. The school VA score juxtaposes the actual level of achievement attained by students attending a certain school with the level of achievement that is expected from students who have the same background characteristics. If these background characteristics affect students’ achievement, including these variables as covariates in the VA model—thereby statistically controlling for them—will help to make the VA scores as unbiased as possible (e.g., Ehlert et al., 2014; Timmermans et al., 2011).

In order to estimate causal effects of school effectiveness (i.e., VA scores) based on observational data, we followed the suggestion from Steiner et al. (2010) for scenarios in which there is no complete knowledge of the covariates that might have an influence in the selection process (i.e., in our case, the process of how students are selected by or sorted to different schools). More specifically, they recommend to consider theoretical, empirical, and expert knowledge in the selection process in order to allow for an identification of the most crucial covariates that help to approximate the selection process while at the same time not wasting resources by contemplating covariates without any proven influence. Thus, on the basis of models of school learning (e.g., Haertel et al., 1983; Wang et al., 1993) and findings on predictors of students’ achievement (e.g., Casillas et al., 2012; Genesee et al., 2005; Jansen & Stanat, 2015; Sirin, 2005; Voyer & Voyer, 2014), we chose investigating four sets of covariates in 4: prior achievement in the same domain as the achievement outcome measure, prior achievement in a different domain, sociodemographic and sociocultural background, and motivational variables. In the following, for each of these sets of covariates, we will present an overview of empirical evidence that may support their use in school VA models (but see, e.g., Ehlert et al., 2016; Perry, 2016; Sloane et al., 2013 for discussions on covariate choice in school VA models from a more political point of view).

### Prior academic achievement

Most authors seem to agree that prior academic achievement should be included in school VA models (e.g., Levy et al., 2019; Casillas et al., 2012; Hanushek, 1971; Ray, 2006). One exception is a study on 4- and 5-year-old students (Luyten et al., 2009) that suggested that it is possible to estimate school effects with cross-sectional data (i.e., without controlling for prior achievement).

Until now, most studies on school VA modeling have estimated VA models with prior achievement from the same domain as the outcome variable or used a composite score of achievement (e.g., Muñoz-Chereau & Thomas, 2016; Timmermans et al., 2011). One exception is a longitudinal study with data from 60,000 to 70,000 students from Australia (Marks, 2017). In that study, school VA models controlling for prior achievement in the same domain were compared with school VA models without prior achievement and school VA models with prior achievement in “all” domains (i.e., numeracy and four different subdomains of language). The author concluded that studies should control for prior achievement in different domains when estimating school VA scores in a language domain but that the school VA model controlling only for prior achievement in the same domain was the preferred one for math achievement. The latter recommendation is in contrast to the “medium function hypothesis” (Peng et al., 2020, p. 2) stating that language has a causal influence on mathematical learning. This hypothesis was supported by findings from a recent meta-analysis, indicating that prior language achievement is predictive for later math achievement and that prior math achievement is predictive for later language achievement (Peng et al., 2020).

### Sociodemographic and sociocultural background variables

Most studies on sociodemographic and sociocultural variables in VA research have focused on socioeconomic status (SES), and the resulting conclusions have been mixed. In general, three different positions can be identified in the existing literature. First, some authors reported that across schools, the effect of SES is often encapsulated by prior achievement (Ferrão, 2009; Hægeland & Kirkebøen, 2008). Second, other research indicated that ignoring students’ backgrounds in school VA models may reward or punish the wrong schools (Dearden et al., 2011; Ehlert et al., 2014; Leckie & Goldstein, 2019; Timmermans et al., 2011) and that students’ learning gains were higher in schools with higher SES than in those with lower SES, also after controlling for prior achievement (De Fraine et al., 2003; Dumont et al., 2013). A third position is that the inclusion or exclusion of students’ background variables depends on the purpose of school VA scores (Muñoz-Chereau & Thomas, 2016; Tekwe et al., 2004). To the best of our knowledge, no studies have investigated the inclusion or exclusion of students’ language or migration background in school VA models, and only one study investigated the inclusion of students’ sex, reporting sex effects in favor of girls in some subjects (see Fitz-Gibbon, 1997).

### Motivational variables

To the best of our knowledge, no previous studies have investigated the inclusion of motivational variables. Additionally, most prior studies on VA modeling did not include any motivational variables in their VA models (Levy et al., 2019). However, researchers and practitioners have begun to emphasize the importance of noncognitive skills in students, for example, by highlighting that “soft skills predict success in life, that they causally produce that success, and that programs that enhance soft skills have an important place in an effective portfolio of public policies” (Heckman & Kautz, 2012, p. 451). After the enactment of the Every Student Succeeds Act (Every Student Succeeds Act, 2015), which allowed for more “soft skills” in schools’ curricula (e.g., Gallup Inc., 2018; Pelletier, 2018), these noncognitive student variables might also become more important in accountability systems and more specifically in VA models.

## The present study

There seems to be consensus that school VA models should include measures of prior achievement that come from the same domain as the achievement for which the VA score was estimated. However, it is still not clear whether it should be the only covariate, or whether (and if so, which) other covariates can contribute to school VA scores being as unbiased as possible. Only a few studies have sought to determine which variables should be included in or excluded from VA models on the school level. Most of these previous studies have reported strong positive correlations between school VA scores from different school VA models with different covariates. However, they stressed that strong correlations will not prevent individual schools being misclassified (e.g., Ehlert et al., 2014; Timmermans et al., 2011). The body of research on VA models is still growing, also within the last decade, even though not as fast as it was the case in the earlier 2000s (as reported in a systematic review; Levy et al., 2019). However, while previous studies have compared VA models with different covariates (e.g., Ehlert et al., 2014), to the best of our knowledge, no previous study has systematically investigated different combinations of covariates when estimating school VA scores by studying every possible combination of the chosen covariates. The present study takes important steps toward filling this gap by analyzing how different sets of covariates affect school VA scores for math and language achievement. This is based on the idea to combine theory- and data-driven approaches, by considering covariates that have previously been found to have an influence on students’ achievement and not only including them separately but systematically combine them with each other. This approach is similar to the “specification curve analysis” (e.g., Rohrer et al., 2017; Simonsohn et al., 2015), suggesting to consider every possible model specification and allowing for a systematic investigation.

As school VA scores are, by definition, everything that cannot be explained by the covariates included in the school VA model, higher amounts of explained variance (i.e., *R*^2^) will reduce the heterogeneity in school VA scores that can be attributed to between-school differences in student intake. Additionally, as VA scores are supposed to provide an estimate of a school’s effectiveness that is as unbiased as possible, independently of students’ backgrounds, an educational system with diverse student populations is best suited for a systematic comparison of VA models. One particularly diverse and multilingual school setting is situated in Luxembourg, a tiny country in the heart of Europe. Luxembourg seems to experience the increase in societal and student diversity even faster than other countries, which can be attributed to, among other factors, its small size, a traditional multilingualism, and an economic system based on immigration. For example, in the 2016–2017 school year, 64% of all newly enrolled students did not speak the first language of instruction (i.e., Luxembourgish) at home (Ministry of National Education, Children and Youth, 2018). This highly diverse school setting poses a challenge for students, teachers, and schools. On the other hand, it offers the opportunity to investigate this unique educational learning environment as an anticipatory model for other educational systems with an increase in student diversity.

Further, many scholars consider VA scores that are applied for consequential decision making to be subject to Campbell’s law (1976), which states that “the more any quantitative social indicator is used for social decision-making, the more subject it will be to corruption pressures and the more apt it will be to distort and corrupt the social processes it is intended to monitor” (p. 49). More concretely, some of the concerns when using school VA scores for high-stakes decisions include psychological consequences for all parties concerned (i.e., teachers, students, and parents) and a risk of attempts of gaming the system (e.g., Foley & Goldstein, 2012; Leckie & Goldstein, 2019). While some researchers still acknowledge the usefulness of teacher VA scores in accountability contexts (Loeb, 2013; Scherrer, 2011), others have indicated the usefulness of an informative use of school VA scores in order to inform teachers, parents, or schools and to learn from those schools that have been classified as “effective” (e.g., Ferrão, 2012; Leckie & Goldstein, 2019). In the present study, we thus use representative longitudinal achievement data obtained from the low-stakes Luxembourg School Monitoring Programme (LUCET, 2021) to circumvent these potential sources of bias that result from pressure due to accountability and explore a more informative use of school VA scores.

Capitalizing on this unique data, we examined school VA scores for two key outcome domains—math and language achievement—using four different sets of covariates that were systematically combined with each other: (a) prior achievement in the same outcome domain (e.g., a measure of prior math achievement to derive the school VA score for math achievement), (b) prior achievement in the other outcome domain (e.g., prior language achievement to derive the school VA score for math achievement), (c) sociodemographic and sociocultural background characteristics, and (d) motivational variables in the same domain (e.g., self-concept in math to derive the school VA score for math achievement). In doing so, we addressed two research questions:

1. How do different covariate sets affect the amount of explained variance by the VA models that are used to calculate school VA scores for math and language achievement?
2. How sensitive is the evaluation of schools to the selection of covariates in the VA model?

## Method

### Participants

For the analyses, we used longitudinal large-scale data obtained from the Luxembourg School Monitoring Programme ÉpStan (LUCET, 2021). The ÉpStan assesses students’ academic competencies (in math and languages), their achievement motivation, as well as information on their sociodemographic and sociocultural backgrounds at the beginning of Grades 1, 3, 5, 7, and 9, respectively. Every year, the entire student population in each of these grade levels participates in the ÉpStan. In the present paper, we used longitudinal data from the student cohort that participated in ÉpStan in Grade 1 in 2014. For our analyses, we included only the *N* = 3603 students attending 153 primary schools who took the math and/or language achievement tests in Grade 3 after participating in Grade 1 in 2014 (see Table 1 for details on the sample composition and excluded students). Excluded students (*N* = 1400) were either absent on the day of testing in third grade (*N* = 1068; e.g., due to illness, because they left the Luxembourgish school system, or because they repeated a grade between first and third grade), or they changed schools between Grades 1 and 3 (*N* = 332). Excluded students had lower achievement values in Grade 1 than included students, indicating that nonparticipation in Grade 3 could most likely be due to repeating a grade between first and third grade. Notably, the longitudinal sample on which the present VA models were based represents almost the entire student population that successfully progressed from Grade 1 in 2014 to Grade 3 in 2016 at the same school. All participating children and their parents or legal guardians were duly informed before data collection and had the opportunity to opt-out. To ensure students’ privacy and in accordance with the European General Data Protection Regulation, a so-called “Trusted Third Party” pseudonymized the data (for more information, see LUCET, 2021). For the present analysis, we used an anonymized data set.

**Table 1:** Details on the sample composition and excluded students

	Included students^a^	Excluded (no participation in Grade 3)	Excluded (students switched school)
Number of students	3603	1068	332
Mean prior math ach. in Grade 1	520 (*SD* = 92)	437 (*SD* = 103)	499 (*SD* = 82)
Mean prior language ach. in Grade 1	519 (*SD* = 93)	441 (*SD* = 103)	489 (*SD* = 85)
Percentage of female students	50%	47%	49%
First language of instruction not spoken at home	49%	65%	60%
Mean HISEI score	49.9 (*SD* = 15.4)	42.6 (*SD* = 15.5)	44.9 (*SD* = 15.2)
Mean math ach. in Grade 3	515 (*SD* = 104)	–	*479* (*SD* = 93)
Mean language ach. in Grade 3	512 (*SD* = 102)	–	474 (*SD* = 103)

*ach*. achievement

^a^Based on the criteria described above

### Measures

#### Academic achievement

Achievement measures were used as outcome variables (i.e., math and language achievement in Grade 3) and as covariates (i.e., math and language achievement in Grade 1). While typically in VA research, the difference between prior and later achievement is only 1 year, the 2-year-gap in Luxembourg is necessary as the primary school system consists of learning cycles of 2 years. Thus, at the beginning of Grade 3, the knowledge gained in the first learning cycle of elementary school is tested, spanning Grade 1 and Grade 2. All achievement measures were assessed with standardized achievement tests, which were developed on the basis of the national curriculum standards (defined by the Ministry of National Education, Children and Youth, 2011) and are used as such in the national school monitoring. All achievement tests (i.e., listening comprehension, reading comprehension, and math) were developed and scaled as one-dimensional tests (Fischbach et al., 2014). The tests were administered in the classroom setting, given in a paper-and-pencil format, and mostly based on closed-format items. To scale the items, a unidimensional Rasch model was used (Fischbach et al., 2014; see Nagy & Neumann, 2010; Wu et al., 2007). Weighted likelihood estimates (Warm, 1989) were used as measures of students’ achievement (Fischbach et al., 2014). The reliability estimates of all achievement scales were calculated using the *TAM* package version 3.3.10 (Robitzsch et al., 2019).

#### Math achievement

The math tests in Grade 3 were constructed in German because the language of instruction in Grades 1 and 2 is German. Math items in Grade 3 assessed children’s competencies in three areas: “numbers and operations,” “space and form,” and “quantities and measures.” As it is typical for large-scale assessments, one global score for math competencies was used (e.g., PISA, OECD, 2018), designed with a mean of 500 and a standard deviation of 100. The reliability of the math test scores in Grade 3 was 0.90. Math achievement in Grade 1 was assessed in Luxembourgish (which is, although politically and culturally a language on its own, linguistically speaking a variety of German, see, Dalby, 1999) because the language of instruction in preschool is Luxembourgish. Mathematics items in Grade 1 assessed children’s competencies in the domains “numbers and operations,” “space and shape,” and “size and measurement.”.^1^ The reliability of the math test scores in Grade 1 was 0.75.

#### Language achievement

Two scales were used to operationalize language achievement in Grade 3: children’s listening and reading comprehension in the German language, each on a scale designed with a mean of 500 and a standard deviation of 100. Listening comprehension was based on the subskills “identifying and applying information presented in a text” and “construing information and activating listening strategies.” Reading comprehension was assessed with the subskills “identifying and applying information presented in a text” and “construing information and activating reading strategies/techniques”.^2^ The reliability of the listening comprehension/reading comprehension test scores in Grade 3 was 0.81/0.88. We computed a mean score across listening and reading comprehension in the German language to represent students’ language achievement in Grade 3. Language achievement in Grade 1 consists of the two scales “early literacy comprehension” and “listening comprehension” in Luxembourgish in Grade 1 because the language of instruction in preschool is Luxembourgish. Listening comprehension was assessed with the two subskills “identifying and applying information presented in a text” and “construing information and activating listening strategies” with different kinds of texts, which were played from an audio CD. Early literacy comprehension was assessed with the subskills “phonological awareness,” “visual discrimination,” and “understanding of the alphabetic principle”.^3^ The reliability of the listening comprehension/reading comprehension test scores in Grade 1 was 0.70/0.70. As these scores were used as independent variables and as reading and listening comprehension cover different subdomains of language achievement (see, e.g., Elgart, 1978; van Zeeland & Schmitt, 2013), both test scores were included in the models.

#### Note on the reliability of achievement measures

As reported above, the weighted likelihood estimates representing students’ domain-specific achievement demonstrated score reliability ranging between 0.70 and 0.90. While depending on the purpose of the application of those measures, especially those reliability measures around 0.70 could be considered as rather low, it has been shown that they suffice research purposes (Schmitt, 1996). Other authors argue that for reliabilities above 0.60, the exact size of the reliability plays a less important role in reducing bias than the nature of the covariates (Steiner et al., 2011, 2015) and the importance of a covariate (i.e., an important variable with a reliability of 0.60 is still better than a perfectly measured poor variable; Cook et al., 2009). In addition, the present domain-specific tests were developed by expert panels (i.e., teachers, content-specialists on teaching and learning, psychometricians) to ensure content validity of all test items. Further, psychometric experts examined all test items for whether they exhibit differential item functioning across student cohorts attending the same grade level to allow for commensurable measures across time.

### Sociodemographic and sociocultural background variables

To obtain information about children’s sociodemographic and sociocultural backgrounds, parents filled out a questionnaire when the students were in Grade 1. Parents were asked to locate their profession within given occupational categories; these categories were based on the International Standard Classification of Occupations. For each occupational category, the average value of the ISEI scale (International Socio-Economic Index of occupational status; see Ganzeboom, 2010) was computed to obtain a proxy for parents’ SES. ISEI values had a mean of 49.9 and a standard deviation of 15.4. Parents were also asked where they and their child were born to indicate their migration status. In the present analyses, migration status was dummy-coded with “native” as the reference category. On the student questionnaire in Grade 1, students were asked to indicate what language(s) they spoke with their father and their mother. As the first language of instruction is Luxembourgish, not speaking any Luxembourgish at home represents a challenge for the newly enrolled students. We thus created a dummy variable to differentiate between students who did not speak any Luxembourgish at home and those who spoke Luxembourgish with at least one parent (reference category). Students’ sex was retrieved from the official database of the Ministry of National Education, Children and Youth.

### Motivational variables

Students in Grade 1 were asked about their domain-specific learning motivation (i.e., academic anxiety, self-concept, and interest) in math and in the German language (which is most closely related to Luxembourgish) on a 2-point rating scale (“agree” and “disagree”). Specifically, domain-specific anxiety was assessed with one item per domain (e.g., “I’m afraid of math,” derived from Gogol et al., 2014); academic self-concept and interest were each assessed with two items per domain (e.g., “I am good at math,” derived from Marsh, 1990, or “I’m interested in the subject of German language,” derived from Gogol et al., 2016). The reliabilities, measured with Cronbach’s alpha, were 0.53 for academic self-concept in math, 0.60 for academic self-concept in the German language, 0.66 in interest in math, and 0.71 for interest in the German language. Additional analyses on their convergent and discriminant validity showed that domain-specific achievement test scores in both Grade 1 and Grade 3 followed the theoretically predicted pattern to academic self-concepts in matching and non-matching domains (Niepel et al., 2017; van der Westhuizen et al., 2019), indicating that the motivational scales measure what they are purported to measure.

### Analysis

We conducted all analyses with R version 3.6.1 (R Core Team, 2019).

#### Data preparation

Due to the inclusion criterion of students’ participation in the achievement tests in Grade 3, there were no missing data in the achievement data in Grade 3. Missing data on the covariates were imputed using multiple multilevel imputation with 20 imputations, 50,000 burn-in iterations, and 5000 iterations between imputations using the *mitml* package version 0.3–7 (Grund et al., 2019) as an interface for the *jomo* package version 2.6–9 (Quartagno & Carpenter, 2019).

#### Between-school differences in student intake

Intraclass correlations, measured with ICC(1) values, estimate how much of the total variance can be attributed to differences between schools in student intake (see, e.g., Bliese, 2000; Lüdtke et al., 2006). ICC(1) was computed with the *rptR* package version 0.9.22. (Stoffel et al., 2019).

#### Multicollinearity

Data was checked for multicollinearity by estimating the variance inflation factor (VIF), using the package *performance* version 0.9.0 (Lüdecke et al., 2021).

#### Estimation of VA scores

Multilevel models were used to estimate the VA scores (for more details, see, e.g., Doran & Lockwood, 2006). We chose them because on the one hand, they represent one of the two most commonly used model types in VA research (the other one being linear regressions; Levy et al., 2019; Kurtz, 2018). On the other hand, in a study with the same sample as the present study, multilevel models prevailed over other models, including linear and nonlinear “classical” and machine learning models, in the estimation of school VA scores (Levy et al., 2020). Thus, the model choice reflects current practices in VA research and applications, and at the same time seems to be the best choice given the present dataset.

Specifically, two-level models (with students located at Level 1 and schools located at Level 2; see Eqs. 1 and 2) with random intercepts were estimated using the *lmer* function from the lme4 package (Bates et al., 2015).

1 $$\begin{array}{cc}\mathrm{Level }1:& {A}_{\mathrm{ij}}={\upbeta }_{0\mathrm{j}}+{{\varvec{\upbeta}}}_{1}{{\varvec{X}}}_{\mathrm{ij}}+{e}_{\mathrm{ij}}\end{array}$$

2 $$\begin{array}{cc}\mathrm{Level }2:& {\upbeta }_{0\mathrm{j}}={\upgamma }_{00}+{\upmu }_{0\mathrm{j}}\end{array}$$

In Eq. 1, *A*_ij_ is the achievement in math or language of student *i* in school *j* in Grade 3. **X**_ij_ is a vector of the different covariates X of student *i* in school *j* as assessed in Grade 1. β_0j_ is the intercept, **β**_**1**_ is a vector of regression coefficients linking the covariates to achievement in Grade 3, and *e*_ij_ is a residual term (assumed to be normally distributed with a mean of zero and a common variance of σ^2^ for all schools). Equation 2 represents between-school differences in the intercept (β_0j_) in Eq. 1. γ_00_ represents the intercept of the outcome variable *A* that is assumed to be constant across all students and schools (i.e., a fixed effect). μ_0j_ is a random residual error term that can vary between schools and was assumed to be normally distributed with a mean of 0 and a variance specified as σ_μ0_^2^ (Hox, 2013). The VA score of a school *j* can be quantified in terms of an estimate of the random effect $\widehat{\upmu }$_0j_ for a particular school at Level 2 (i.e., the residual for a certain school; see Ferrão & Goldstein, 2009). School VA scores were estimated using the *ranef* function from the *lme4* package (Bates et al., 2015).

#### Systematic combination of covariate sets in the VA models

The idea of systematically combining different covariate sets is based on specification curve analysis (Simonsohn et al., 2015; see Rohrer et al., 2017, for an application), which aims to calculate all “reasonable” specifications. Applied to our case, we estimated all school VA scores for math and language achievement that could be derived from all possible combinations of four covariate sets: (a) prior achievement in the same domain, (b) prior achievement in a different domain, (c) sociodemographic and sociocultural background variables, and (d) motivational variables in the same domain. Doing so resulted in 15 (2^4^–1) covariate set combinations per domain, and thus 15 school VA scores in math achievement and 15 school VA scores for language achievement per school, respectively (see Table 2 for more detail).

**Table 2:** Overview of covariate set combinations and the amount of variance explained by the respective covariate sets

Model number	Covariate (sets) included in the VA model				
	Prior math achievement	Prior language achievement	Sociodemographic and sociocultural background^a^	Motivational variables^b^	*R*^2^
School VA score for Math achievement in Grade 3					
1	x				.40
2		x			.26
3	x	x			.43
4			x		.09
5	x		x		.42
6		x	x		.30
7	x	x	x		.45
8				x	.06
9	x			x	.40
10		x		x	.27
11	x	x		x	.43
12			x	x	.13
13	x		x	x	.43
14		x	x	x	.31
15	x	x	x	x	.45
School VA score for language achievement in Grade 3					
1	x				.16
2		x			.35
3	x	x			.36
4			x		.26
5	x		x		.36
6		x	x		.45
7	x	x	x		.46
8				x	.06
9	x			x	.19
10		x		x	.36
11	x	x		x	.37
12			x	x	.29
13	x		x	x	.38
14		x	x	x	.46
15	x	x	x	x	.47

x = Covariate (set) was included in the school VA model

^a^This covariate set comprised gender, SES (as measured by HISEI), first language of instruction spoken at home, and migration background

^b^We used motivational variables (i.e., anxiety, self-concept, and interest measured in Grade 1) in math to estimate the school VA score for math achievement. Likewise, we used motivational variables for language (i.e., anxiety, self-concept, and interest measured in Grade 1) to estimate the school VA score for language achievement

Notably, each model was run for each imputed data set (i.e., resulting in 2 domains * 15 models * 20 imputed data sets = 600 analyses). To evaluate the school VA scores in further analyses, we pooled the domain-specific scores across the 20 imputed data sets to obtain a mean school VA score for each school for a certain combination of covariates.

To address research question 1, we evaluated the amount of explained variance of the underlying VA model in terms of the total amount of variance (*R*^2^s) explained by the covariates. The *R*^2^ values were computed in accordance with Snijders and Bosker (2012, p. 112). Further, we tackled research question 2 on how the evaluation of schools depends on the choice of covariates in the VA models by computing correlations of VA scores as obtained from various school VA models and by analyzing the implications of covariate selection on benchmark classifications. Specifically, we used benchmarks classifying schools below the 25th percentile as “needs improvement,” schools between the 25th and 75th percentiles as “moderately effective,” and schools above the 75th percentile as “highly effective” (e.g., Marzano & Toth, 2013). We calculated consistency scores based on the benchmark classifications from the school VA model that included all covariates, as this allowed to analyze the impact that the exclusion of the different covariate sets can have. More specifically, for every school VA model, the percentage of schools identified at the same benchmark classification as the one in the model that included all covariates was used as a measure of consistency, where higher values represented a higher concordance with the benchmark classifications from the school VA model that included all of the covariates. In addition, we calculated percentages of disagreement for benchmark classifications of all school VA scores with each other and “disagreement” was defined as schools being placed at a different benchmark (i.e., “needs improvement,” “moderately effective,” or “highly effective”). The practical implications on individual schools will additionally be illustrated on the example of five schools that were randomly chosen as examples for high ranges in VA scores (schools 1 and 2), and for constant high, low, or medium VA scores (schools 3, 4, and 5), respectively (see Table 8 for descriptive data on these schools). The same example schools were used in Levy et al., (2020).

## Results

### Preliminary analyses of between-school differences in student intake

Table 3 shows the intraclass correlations, computed as ICC(1) values, and variance inflation factors (VIF), as an estimate of multicollinearity. For all achievement variables, parents’ SES, and “Luxembourgish spoken at home,” the ICC(1) was greater than 0.1, indicating that a substantial proportion of the total variance could be attributed to mean differences between schools when students began primary school in Grade 1. Furthermore, all VIF values, exemplified in Table 3 based on the school VA model with math achievement as a dependent variable and all covariate sets included, were smaller than 5, indicating that no multicollinearity is present in the data (James et al., 2013).

**Table 3:** Intraclass correlations and variance inflation factors of the different covariates

Variable	ICC(1)	VIF^a^
Prior math achievement	.11	1.54
Prior reading achievement	.14	1.59
Prior listening achievement	.15	1.45
Gender	.00	1.05
Socioeconomic status	.12	1.10
Luxembourgish spoken at home	.11	1.87
Anxiety in math	.07	1.05
Self-concept in math	.03	1.07
Interest in math	.03	1.06
Anxiety in language	.04	/
Self-concept in language	.03	/
Interest in language	.03	/

ICC(1) = intraclass correlation calculated as τ^2^/(τ^2^ + σ^2^), with τ^2^ = variance between schools; σ^2^ = variance within schools. VIF = variance inflation factor

^a^Exemplified with the 20th imputed model with all covariates included into the model with math achievement as a dependent variable

### Research question 1: how do the different covariate sets affect the amount of explained variance by the VA models?

Table 2 shows the amount of explained variance (*R*^2^) from the 15 different covariate sets for the school VA scores in math and language, respectively. In the following, results will be described and in the discussion section, they will be put into context and possible explanations will be discussed.

#### School VA models for math achievement

As expected, the highest amount of explained variance (*R*^2^ = 0.45) was obtained when all four covariate sets were included (model 15). Nevertheless, some covariates turned out to be more important than others. More specifically, the amount of explained variance was considerably higher when prior math achievement was included (i.e., models 1, 3, 5, 7, 9, 11, 13, 15), both when considering the range (from 0.40 to 0.45) and the median (0.43), as compared with its exclusion (i.e., models 2, 4, 6, 8, 10, 12, 14; ranging from 0.06 to 0.31; *Mdn* = 0.26). The *R*^2^s were higher and had a smaller range when prior language achievement was included (0.26 to 0.45) in comparison to its exclusion (0.06 to 0.43), even though the median was lower for the models with prior language achievement (0.37 vs. 0.40). The range in *R*^2^s was slightly higher (by 0.02) when the sociodemographic and sociocultural background variables were included (0.09 to 0.45), but the median was lower (0.37) in comparison to their exclusion (0.06 to 0.43; *Mdn* = 0.40). Both the range and the median for the *R*^2^ were lower when the motivational variables were included (0.06 to 0.45; *Mdn* = 0.36) as compared to their exclusion (0.09 to 0.45; *Mdn* = 0.40).

#### School VA models for language achievement

As expected, the highest amount of explained variance (*R*^2^ = 0.47) was obtained when all four covariate sets were included. The differences in *R*^2^s between the inclusion of prior language achievement (ranging from 0.35 to 0.47; *Mdn* = 0.41) and its exclusion (0.06 to 0.38; *Mdn* = 0.26) were substantial. However, the range and median of explained variance were only slightly higher when prior math achievement was included (0.16 to 0.47; *Mdn* = 0.37) than when it was excluded (0.06 to 0.46; *Mdn* = 0.35). Bigger differences in the range and median could be observed when the sociodemographic and sociocultural background variables were included (0.26 to 0.47; *Mdn* = 0.41) in comparison with when they were excluded (0.06 to 0.37; *Mdn* = 0.35). The amount of explained variance when the motivational covariates were included (0.06 to 0.47; *Mdn* = 0.37) was very similar to the amount of explained variance when they were excluded (0.16 to 0.46; *Mdn* = 0.36).

### Research question 2: how sensitive is the evaluation of schools to the selection of covariates in the VA model?

#### Descriptives and correlations between school VA scores

##### School VA models for math achievement

Table 4 shows the minimum and maximum values, median, and standard deviation for VA scores resulting from the different VA models for math achievement. The mean is not depicted, as it is 0 by definition in any case. When interpreting the size of median and standard deviation, it should be considered that the scale on which the dependent variables for the calculation of VA scores are based was designed to have a mean of 500 and a standard deviation of 100.

**Table 4:** Correlations between the school VA scores for Math achievement as obtained from the VA models using different covariate sets

Model Nr	Min	Max	Median	*SD*	1	2	3	4	5	6	7	8	9	10	11	12	13	14
1	− 62	75	− 0.56	23.7	–													
2	− 78	87	− 0.13	25.4	.84	–												
3	− 71	75	− 0.57	24.7	.97	.91	–											
4	− 48	53	0.38	20.7	.78	.83	.73	–										
5	− 61	67	0.77	21.4	.99	.84	.97	.77	–									
6	− 72	87	− 1.62	23.9	.83	.99	.91	.80	.85	–								
7	− 74	74	− 0.35	23.6	.95	.90	.99	.70	.97	.92	–							
8	− 65	65	− 0.39	26.0	.79	.79	.71	.96	.74	.74	.66	–						
9	− 62	73	− 0.96	23.4	1.00	.84	.97	.77	.98	.82	.95	.79	–					
10	− 76	83	− 0.99	24.4	.85	.99	.92	.82	.85	.98	.91	.80	.85	–				
11	− 71	73	− 0.81	24.4	.97	.91	1.00	.72	.97	.91	.99	.71	.97	.92	–			
12	− 45	52	1.22	19.3	.80	.83	.75	.98	.78	.80	.72	.97	.80	.84	.75	–		
13	− 62	65	0.02	21.2	.98	.84	.97	.76	1.00	.84	.97	.74	.99	.85	.97	.79	–	
14	− 71	84	− 1.53	23.2	.83	.98	.92	.79	.85	1.00	.92	.75	.84	.99	.92	.81	.86	–
15	− 74	72	− 0.32	23.4	.94	.90	.99	.70	.97	.91	1.00	.67	.95	.91	.99	.73	.97	.92

*Nr* number, *Min*. minimum value of school VA scores, *Max*. maximum value of school VA scores, *SD* standard deviation of school VA scores. Details on covariate inclusion in the respective models can be seen in Table 2

In addition, Table 4 shows the correlations between the school VA scores resulting from the different models, ranging from.66 to 1.00 (*Mdn* = 0.91). When prior math achievement was included in the VA models, the correlations between the VA scores ranged from 0.94 to 1.00 (*Mdn* = 0.98). However, when prior math achievement was not included, the range of the correlations between the VA scores was considerably broader (0.74 ≤ *r* ≤ 1.00). Additionally, the only correlations that were equal to 1.00 were those between a model with motivational variables and the same model without the motivational variables (e.g., *r* = 1.00 for the correlation between model 9, which included prior math achievement and motivation, and model 1, which included only prior math achievement).

##### School VA models for language achievement

A similar pattern of results was obtained for school VA scores in the language domain (Table 5). Overall, the correlations ranged from 0.81 to 1.00 (*Mdn* = 0.92). When prior achievement in language was included in the VA models, the correlations between the VA scores ranged from 0.96 to 1.00 (*Mdn* = 0.98). When both prior language achievement and sociodemographic characteristics were included, the correlations between the school VA scores were even higher (0.99 ≤ *r* ≤ 1.00; *Mdn* = 1.00). Additionally, any correlations equal to 1.00 were observed either between a model with the motivational variables (e.g., model 9) and the same model without the motivational variables (e.g., model 1) or between a model with prior math achievement (e.g., model 3) and the same model without prior math achievement (e.g., model 2).

**Table 5:** Correlations between the school VA score for language achievement as obtained from the VA models using different covariate sets

Model Nr	Min	Max	Median	*SD*	1	2	3	4	5	6	7	8	9	10	11	12	13	14
1	− 109	101	2.08	36.6	–													
2	− 108	83	4.54	30.4	.90	–												
3	− 105	79	3.40	30.4	.92	1.00	–											
4	− 81	56	0.30	27.1	.91	.83	.82	–										
5	− 72	67	2.14	25.4	.95	.88	.89	.95	–									
6	− 80	57	1.46	23.4	.86	.97	.97	.85	.91	–								
7	− 77	52	0.69	23.5	.88	.97	.97	.85	.93	.99	–							
8	− 110	75	2.42	38.8	.96	.86	.85	.94	.90	.81	.81	–						
9	− 107	93	2.38	35.7	1.00	.90	.92	.90	.95	.86	.88	.96	–					
10	− 109	79	4.92	30.0	.90	1.00	1.00	.82	.88	.97	.96	.86	.91	–				
11	− 106	76	4.6	30.1	.92	.99	1.00	.82	.89	.96	.97	.86	.92	1.00	–			
12	− 77	56	1.63	26.0	.91	.83	.83	.99	.95	.86	.85	.95	.92	.84	.83	–		
13	− 71	63	2.23	25.0	.95	.87	.89	.94	1.00	.90	.93	.91	.95	.88	.90	.95.2	–	
14	− 81	55	1.33	23.1	.86	.97	.97	.85	.91	1.00	.99	.82	.87	.97	.97	.86	.91	–
15	− 78	51	.91	23.3	.88	.97	.97	.85	.93	.99	1.00	.81	.88	.97	.97	.86	.93	.99

*Nr* number, *Min*. minimum, *Max*. maximum, *SD* standard deviation. Details on covariate inclusion in the respective models can be seen in Table 2

#### Implications on benchmark classifications

The benchmark classifications based on the school VA scores resulting from VA models with different covariate sets can be substantially different from each other, with percentages of disagreement ranging from 1.3 to 39.9% (Table 6, math achievement as a dependent variables) and from 1.3 to 33.3% (Table 7, language achievement as a dependent variable), respectively. A more detailed analysis of the number of schools classified at a certain benchmark by the different models, in comparison with the number of schools classified by the VA model with all four covariate sets included, can be found in the Online Resource 1 for the math VA models and Online Resource 2 for the language VA models.

**Table 6:** Average percentage of disagreement between benchmark classifications based on the different school VA models with math achievement as a dependent variable

Model Nr	Prior math ach	Prior language ach	Background^a^	Motivation^b^	1	2	3	4	5	6	7	8	9	10	11	12	13	14
1	x				–													
2		x			24.8	–												
3	x	x			13.1	20.9	–											
4			x		29.4	28.8	37.3	–										
5	x		x		10.5	25.5	11.8	30.1	–									
6		x	x		31.4	10.5	23.5	30.7	29.4	–								
7	x	x	x		17.0	20.9	5.2	36.6	11.8	22.2	–							
8				x	32.7	34.6	37.9	17.0	37.3	39.2	38.6	–						
9	x			x	2.6	26.1	14.4	29.4	11.8	32.7	18.3	32.7	–					
10		x		x	26.1	3.9	20.9	26.1	26.8	11.8	20.9	32.0	24.8	–				
11	x	x		x	13.1	20.9	3.9	34.6	9.2	22.2	5.2	37.3	14.4	20.9	–			
12			x	x	32.0	33.3	36.6	11.8	30.7	36.6	34.6	11.8	32.0	30.7	34.6	–		
13	x		x	x	13.1	28.1	11.8	31.4	5.2	29.4	10.5	37.3	10.5	25.5	9.2	32.0	–	
14		x	x	x	28.8	13.1	22.2	30.1	28.1	7.8	19.6	35.9	27.5	10.5	20.9	32.0	24.2	–
15	x	x	x	x	18.3	20.9	6.5	37.9	13.1	22.2	1.3	39.9	19.6	20.9	5.2	35.9	11.8	19.6

x = Covariate (set) was included in the VA model; *Nr* number, *ach*. achievement

^a^Sociodemographic and sociocultural background, which comprised gender, SES (as measured with the HISEI), first language of instruction spoken at home, migration background

^b^Motivational variables related to language

**Table 7:** Average percentage of disagreement between benchmark classifications based on the different school VA models with language achievement as a dependent variable

Model Nr	Prior math ach	Prior language ach	Background^a^	Motivation^b^	1	2	3	4	5	6	7	8	9	10	11	12	13	14
1	x				–													
2		x			19.6	–												
3	x	x			18.3	1.3	–											
4			x		17.0	23.5	24.8	–										
5	x		x		15.7	26.1	26.1	13.1	–									
6		x	x		24.8	9.2	10.5	24.8	23.5	–								
7	x	x	x		24.8	10.5	10.5	26.1	20.9	3.9	–							
8				x	13.1	27.5	27.5	19.6	24.8	29.4	31.4	–						
9	x			x	2.6	18.3	17.0	18.3	18.3	23.5	23.5	13.1	–					
10		x		x	18.3	1.3	2.6	22.2	24.8	9.2	10.5	26.1	17.0	–				
11	x	x		x	17.0	2.6	1.3	23.5	24.8	10.5	10.5	26.1	15.7	1.3	–			
12			x	x	17.0	26.1	26.1	6.5	11.8	24.8	23.5	19.6	18.3	24.8	24.8	–		
13	x		x	x	14.4	24.8	23.5	15.7	5.2	22.2	19.6	23.5	15.7	23.5	22.2	13.1	–	
14		x	x	x	26.1	10.5	10.5	26.1	20.9	5.2	3.9	33.3	24.8	10.5	10.5	23.5	19.6	–
15	x	x	x	x	24.8	10.5	10.5	27.5	22.2	5.2	2.6	31.4	23.5	10.5	10.5	24.8	20.9	2.6

x = Covariate (set) was included in the VA model; *Nr* number, *ach*. achievement

^a^Sociodemographic and sociocultural background, which comprised gender, SES (as measured with the HISEI), first language of instruction spoken at home, migration background

^b^Motivational variables related to language

**Fig. 1:**
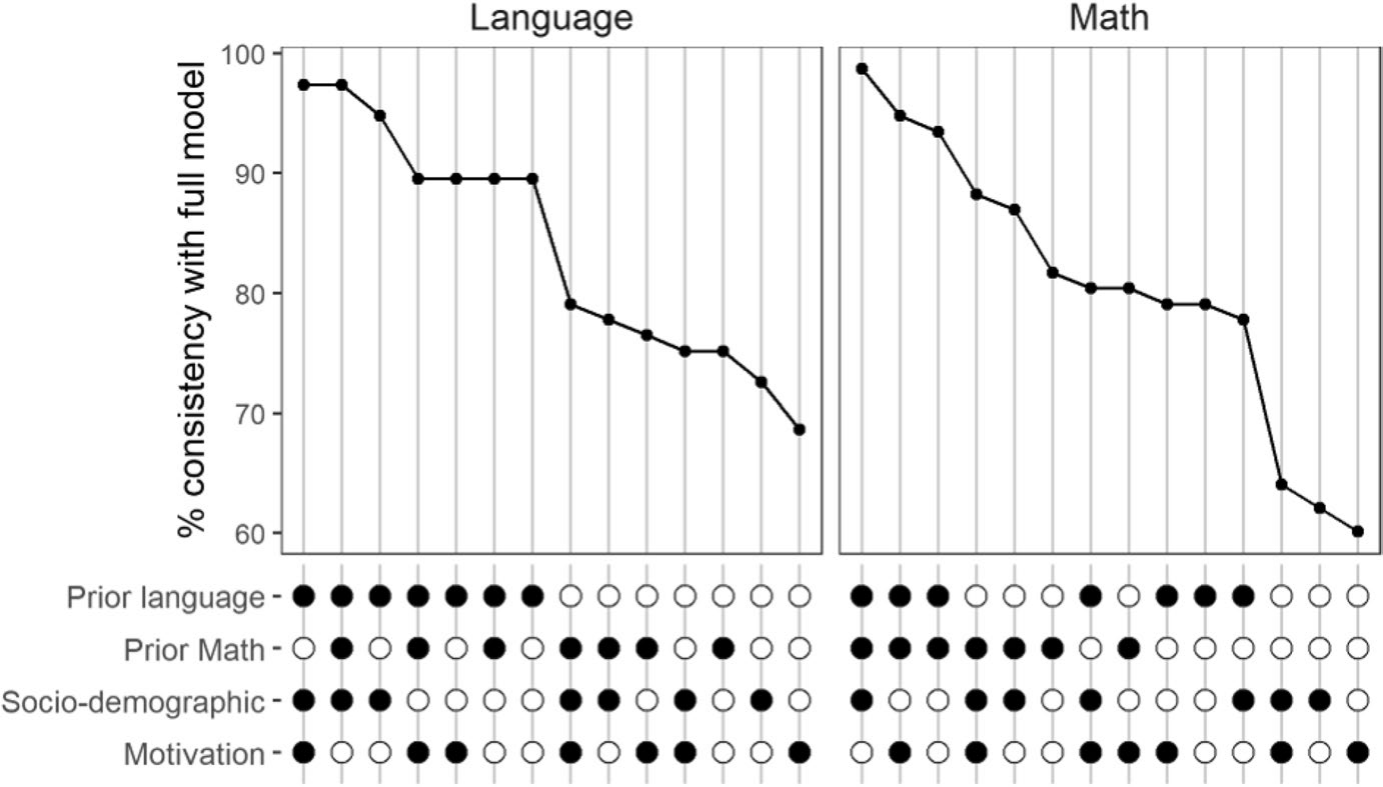
Consistency measures of benchmark classifications as compared with the classifications made by the model that included all of the covariates. Consistency measures for school VA scores in math are shown on the right and school VA scores for language on the left. Below the plots, the color of the dots indicates the inclusion (black) or exclusion (white) of the respective covariate sets

A more comprehensive overview of the most relevant comparison can be seen in Fig. 1, showing the consistency of the benchmark classifications in comparison to the model that included all four covariate sets (Table 8).

**Table 8:** Descriptive data from the five example schools shown in Fig. 2

	School 1	School 2	School 3	School 4	School 5
Number of students	18	52	33	49	26
Mean prior math achievement in Grade 1	459	575	509	592	534
Mean prior language achievement in Grade 1	468	567	511	587	506
Percentage of female students	33%	54%	61%	53%	58%
Percentage of “First language of instruction not spoken at home”	66%	33%	55%	80%	46%
Mean HISEI score	41.0	56.2	54.6	56.8	48.2
Mean math achievement in Grade 3	504	540	543	498	517
Mean language achievement in Grade 3	476	579	527	470	510

### School VA models for math achievement

The right side of Fig. 1 shows that the highest consistency measures were reached when prior math and prior language achievement were included, and the lowest values of consistency were reached when neither of these covariate sets were included. In contrast, there seemed to be only small to not observable advantages of including the sociodemographic and sociocultural background variables or motivational variables.

### School VA models for language achievement

The left side of Fig. 1 shows that the highest consistency values were reached when prior language achievement and students’ sociodemographic and sociocultural backgrounds were included, and the lowest values were reached without prior language achievement, whereas there seemed to be no observable advantage of including prior math achievement or motivational variables.

### Real-life implications of choice of covariate for the school VA percentiles

**Fig. 2:**
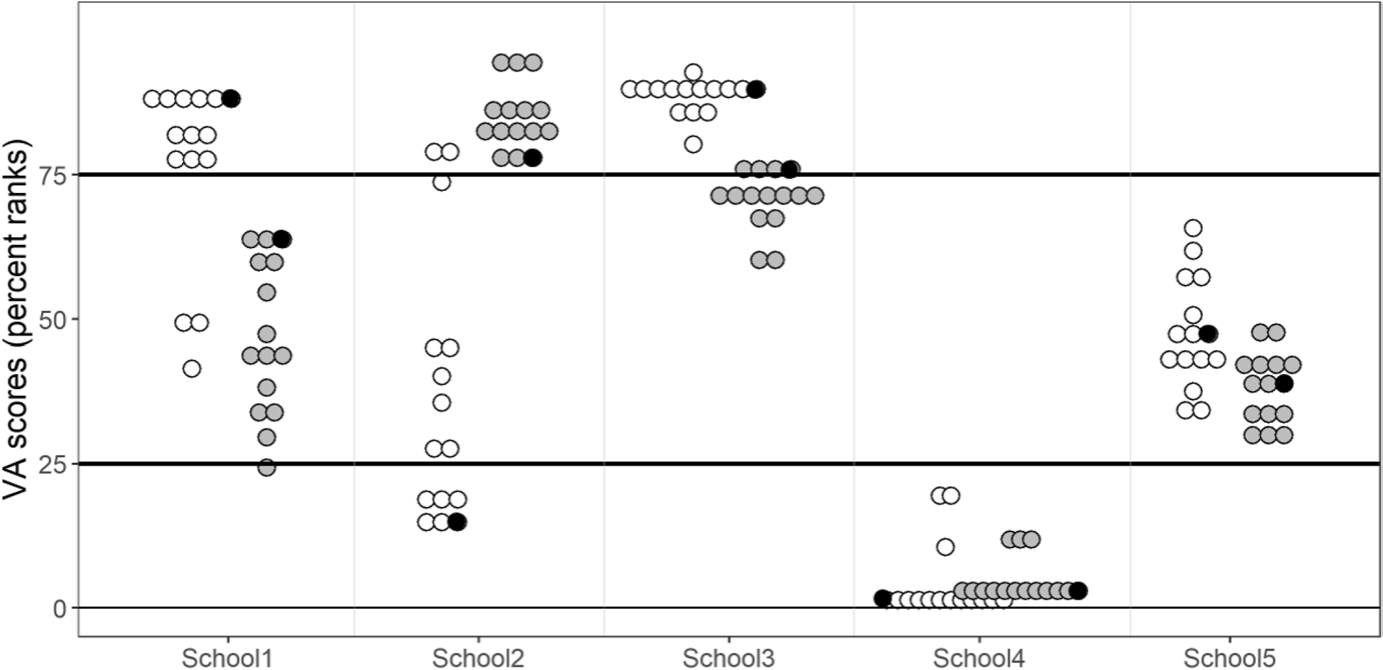
Range of percentiles resulting from math (white) and language (gray) VA scores for five example schools. Every dot represents the school VA percentile as obtained from a certain VA model. The VA models with all the covariates included are marked in black. At the 25^th^ and 75^th^ percentiles, there are cut-off lines to define the border between schools classified as “needs improvement,” “moderately effective,” and “highly effective”

Figure 2 illustrates the real-life implications that the use of different VA scores may have on the example of five schools. It shows the range of the VA percentiles for these schools and illustrates that the effectiveness of some schools may be evaluated quite differently depending on the model used to estimate the school VA scores, whereas other schools have VA scores within a more consistent range (e.g., School 4). The school VA model including all covariate sets is marked in black. For schools 1, 2, and 3, it can even be seen that the inclusion or exclusion of certain covariates can lead to different benchmark classifications. For example, School 1 would be classified as “highly effective” by VA percentiles resulting from those math VA models that included prior math and/or language achievement, whereas it would be classified as “moderately effective” without these covariates (see Online Resource 3 for detailed values of the percentiles that resulted from the respective school VA models).

## Discussion

The VA score of a certain school juxtaposes the actual level of achievement attained by students attending a certain school with the achievement level that is expected from students who have the same background characteristics (e.g., prior achievement or sociodemographic and sociocultural backgrounds). If these characteristics affect students’ achievement, including these variables as covariates in the VA model—thereby statistically controlling for them—may substantially contribute to improving the consistency of VA models (e.g., Ehlert et al., 2014; Timmermans et al., 2011). As there seems to be no final consensus on which covariates should be used in VA models, the aim of the present paper was to systematically analyze and compare different combinations of covariates in school VA models. Of course, not every combination of covariates is as likely to be used in practice. However, in order to conduct a systematic exhaustive comparison and contribute to a cumulative empirical body of knowledge, every possible combination of covariates was considered. In doing so, the present paper addressed two research questions:

1. How do different covariate sets affect the amount of explained variance of the VA models that are used to calculate school VA scores for math and language achievement?
2. How sensitive is the evaluation of schools to the selection of covariates in the VA model?

In the following, we discuss the implications from the results of the math and language school VA models together because the focus of the present paper is on the choice of covariates rather than on the dependent variables.

### How do different covariate sets affect the amount of explained variance of VA models?

As school VA scores include, by definition, everything that cannot be explained by the covariates included in the school VA model, aiming to explain a large amount of variance will reduce the heterogeneity in school VA scores that can be attributed to between-school differences in student intake. More specifically, higher amounts of explained variance in the underlying model that is used to estimate school VA scores will result in less noise in VA scores and thus estimated VA scores that are closer to the “real” school VA score (which could of course be estimated only if all factors causing bias are known and controlled for). While complex models are prone to overfit, the risk is rather low in multilevel models with only a few covariates and a big sample size, as it is the case in 4, which is why we assume that higher amount of explained variance will lead to less bias in VA scores.

In line with previous research on academic achievement, we found that the best predictor of later achievement is prior achievement in the same domain (e.g., Aubrey et al., 2006; Hemmings & Kay, 2010; Reynolds, 1991; Yates, 2000). This supports one of the most prominent VA applications ( “Educational Value-Added Assessment System,” which was built on the Tenessee Value-Added Assessment System, Sanders & Horn, 1994).

Whether prior academic achievement in a different domain affects the amount of explained variance of school VA models depends on the outcome variable. On the one hand, prior achievement in language contributed to explaining additional variance in later math achievement, confirming that early language abilities are important for learning math (Peng et al., 2020), particularly in the multilingual and diverse context of Luxembourgish schools (e.g., Van Rinsveld et al., 2015). On the other hand, the inclusion or exclusion of prior math achievement did not make a substantial difference in explaining language achievement in Grade 3, contradicting findings from a recent meta-analysis (Peng et al., 2020).

Similarly, whether students’ sociodemographic and sociocultural backgrounds affect the amount of explained variance of school VA models depends on the outcome variable. Even though it was not the case in school VA models for math, including students’ sociodemographic and sociocultural background variables in the school VA models for language led to higher amounts of explained variance, which is in line with previous research on the relationship between achievement and sociodemographic variables (e.g., SES, Sirin, 2005; or sex, Voyer & Voyer, 2014). These findings suggest that students’ sociodemographic and sociocultural background variables play crucial roles in the development of language abilities, even when controlling for prior language achievement. It would be interesting for future research to investigate whether the importance of students’ sociodemographic and sociocultural background variables can be replicated in a linguistically more homogenous sample.

Motivational variables seem to incrementally explain variance in students’ achievement in addition to prior achievement (e.g., Marsh & Craven, 2006; Marsh et al., 2005; Valentine et al., 2004), even after cognitive ability was controlled for (Spinath et al., 2006; Steinmayr & Spinath, 2009). Our opposed findings could be explained by the fact that students in Grade 1 reported a high level of motivation in general and between-school differences in motivation (see Table 3) were rather small. Thus, this restriction of range in the between-school differences of motivational measures may have mitigated the possibility that these measures help to improve precision of VA scores. Further, a more recent study indicated that reciprocal effects between motivation (e.g., self-concepts) and achievement are not present in the early years of primary school (Weidinger et al., 2019). One reason may be that many students at that age may not have reached a sufficient level of self-reflection to provide accurate self-reports on how motivated they are in a certain school subject. Future research on VA is required to investigate whether motivational variables play a more important role in improving the amount of explained variance of school VA models in older students.

### How sensitive is the evaluation of schools to the selection of covariates in the VA model?

The investigation of the sensitivity of schools’ evaluations to covariate selection was twofold. First, on a descriptive level, the range, median, standard deviation, and correlations between school VA scores resulting from the school VA models with different combinations of covariates were calculated. Second, implications for the benchmark classifications were investigated across all schools and on an illustrative example involving five schools.

Correlations from different school VA models were moderate to high (ranging from 0.66 to 1.00 in the math VA models and from 0.81 to 1.00 in the language VA models), which is in line with findings from previous studies on teacher and school VA (e.g., Ehlert et al., 2014). These correlations were even higher when considering only the school VA models that contained prior achievement in the same domain (0.94 ≤ *r* ≤ 1.00 for school VA models in math and 0.96 ≤ *r* ≤ 1.00 for school VA models in language). These high correlations between school VA scores could lead to the erroneous conclusion that, for the sake of specifying the most parsimonious school VA model, including prior achievement in the same domain should be enough to obtain consistency in the school VA models. However, as other authors have also stated (e.g., Ehlert et al., 2014 on teacher and school VA; Johnson et al., 2015 on teacher VA; and Timmermans et al., 2011 on school VA), strong correlations will not prevent misclassifications, a phenomenon that will be discussed in more detail below.

The same covariate sets as identified under Research Question 1 led to higher levels of consistency and lower levels of disagreements in the benchmark classifications of school VA scores. In line with most current VA practices (for an exception, see, Luyten et al., 2009), it was crucial to include prior academic achievement in the same domain to get high consistency measures in school VA scores. Further, recommendations from an Australian study on school VA (Marks, 2017) to only control for prior math achievement in the math VA models but to include prior achievement in different domains in the language VA models was not empirically supported in our study. This might be explained by the fact that the sample used by Marks (2017) is more homogenous than our sample and that language(s) spoken at home was never included in his models. The effect of students’ sociodemographic and sociocultural background variables seemed to be encapsulated by prior achievement in the school VA models for math (in line with, e.g., Ferrão, 2009; Hægeland & Kirkebøen, 2008). but not in the school VA models for language, indicating that the exclusion of students’ sociodemographic and sociocultural backgrounds could reward or punish the wrong schools (e.g., Dearden et al., 2011; Timmermans et al., 2011). In the school VA models for both math and language, motivational variables did seem to have an influence on the consistency of the school VA scores, empirically underscoring the decision of most previous studies not to include motivational variables in the VA models (Levy et al., 2019). However, these results might be different in data from the USA, which has added more “soft skills” to schools’ curricula and accountability systems after the enactment of the Every Student Succeeds Act (2015).

The implications on benchmark classifications were also illustrated with five example schools, showing that for individual schools, the range of VA percentiles could change so dramatically that they might be classified as “highly effective” or “needs improvement,” depending on which covariate sets were included. This indicates yet again how crucial covariate selection is when estimating school VA scores. However, it should be kept in mind that there are many more internal and external factors that could come into play but cannot be influenced by schools, such as summer learning losses or mental support at home (Braun, 2015; Darling-Hammond, 2015). Future research on VA models should even be more careful in this regard, as during the current COVID-19 pandemic children were being home schooled for months, which could lead to a higher proportion of influence of students’ backgrounds (see, e.g., Cachón-Zagalaz et al., 2020 for a systematic review on the effects of the pandemic on children).

### Recommendations for educational practice

The findings from the present study support current practice in VA modeling in the sense that prior achievement is crucial in the inclusion of school VA models. However, the present results also indicate that evaluation systems that include only prior achievement from the same domain in school VA models may draw the wrong conclusions about schools’ effectiveness, highlighting the importance of adding additional covariates (e.g., sociodemographic background), as has already been concluded by research on teacher VA (Koedel et al., 2015). Most importantly, given the fluctuation of school VA scores depending on the chosen covariates, the awareness of model differences is essential when applying or interpreting school VA models. In other words, the present results cast further doubt on the use of VA scores for accountability purposes because the evaluation of a certain school’s effectiveness varied widely depending on the covariate set that was chosen, also calling for caution concerning a causal interpretation of school VA scores. Under the assumption that the size of school VA scores can be interpreted as a causal educational effectiveness of these schools, of course reducing the bias in VA scores is of utmost importance. However, previous authors argued that VA scores should not be seen as causal estimates (and rather as descriptives), “except under extreme and unrealistic assumptions” (Rubin et al., 2004, p. 113). As was also shown by Reardon & Raudenbush (2009), a causal interpretation of school VA scores would be based on assumptions, which are implausible in most educational contexts. More specifically, the assumptions that students could potentially be assigned to any school and that school assignments of other students do not have an influence on students’ achievement are both implausible for the present sample, as students usually go to the primary school closest to their home. Furthermore, even though the assumption that a school that is effective for a subgroup would be effective for any other subgroup of students cannot be empirically proven, a recent large-scale international study indicates that there are small but meaningful differences between schools concerning their students’ motivational-affective variables (Brunner et al., 2018). Additionally, an empirical verification of the causality of school VA scores would only be possible through the tautological investigation whether schools with high VA scores lead to higher growth in their students’ achievement, analogously to the commonly known definition of intelligence as “the tests test it” (Boring, 1923).

In line with these recommendations on descriptive rather than causal interpretations of school VA scores, we recommend using VA models with caution and applying VA scores for informative purposes rather than as a mean to base accountability decisions upon (see also, e.g., Floden, 2012; Leckie & Goldstein, 2019). For example, even though only a few schools have constant VA percentiles, these schools could be used as a starting point for further analyses, for example, by comparing the pedagogical strategies of those consistently classified as “highly effective” to those consistently classified as “needs improvement.” Furthermore, the model selection process should include different specifications in order to obtain ranges of potential VA scores instead of one single “true” value of effectiveness (as the one true value does not exist, e.g., Amrein-Beardsley & Holloway, 2017; Conaway & Goldhaber, 2018). In addition, as these estimations are based on human data, they can never be perfectly exact. There are so many more internal and external factors that could come into play but are usually not assessed and/or cannot be influenced by teachers or schools, such as, to name only a few, summer learning losses, mental support at home, medical care, or community environment (Braun, 2015; Darling-Hammond, 2015). School VA scores should thus be considered as one tool to evaluate schools, which are suited rather for pedagogical purposes than for summative evaluations.

## Limitations

Given that so far there is no final consensus on the type of model that should be used to estimate VA scores, we used multilevel models, one of the two most common types of models (next to linear regressions) for calculating VA scores in the international literature (Levy et al., 2019; Kurtz, 2018). As multilevel models directly take into account the hierarchical structure of the data (the fact that students are nested in schools, meaning that students within the same school tend to be more similar to each other than students from different schools) when estimating VA scores, we decided to use multilevel models rather than linear regression models. Furthermore, in a study with the same sample as the present study, multilevel models prevailed over other models, including linear and nonlinear “classical” and machine learning models, in the estimation of school VA scores (Levy et al., 2020). Of course, other types of models would be possible, too. For example, Bayesian models have started to find their way into VA research (in VA research often called “empirical Bayes”; for an overview, see, Guarino et al., 2015). However, in the present paper, we decided against the use of empirical Bayes estimates, as Guarino et al. (2015) concluded that they do not perform well if no random assignment to classes is given (see also, e.g., Kruschke & Liddell, 2018 for an overview on Bayesian statistics; and Kane et al., 2013; or Rothstein, 2009 on random assignment of students and its effect on teacher VA). However, as the term “empirical Bayes” is used quite broadly, it could also be argued that the estimation process in the multilevel model can also be seen as an empirical Bayes technique (Bates, 2009). In addition, in classical models, measurement error cannot be separated from error variance, which is why latent models, such as structural equation models, might be better suited for measurement error in the variables included in the models (see, e.g., Pohl & Carstensen, 2012; and for VA literature on measurement error see, e.g., Ferrão & Goldstein, 2009; Koedel et al., 2012).

Furthermore, only the main effects of the covariates were part of the VA models. In future studies, including interaction effects between covariates in the VA models might allow deeper insights into differences between model specifications in the estimation of VA scores.

For the interpretation of our results, we assumed that higher amount of explained variance will lead to less bias in VA scores. However, it should be noted that, in order to quantify the amount of bias, a validation against credible causal estimates would be necessary, which is not possible given the data of the present study, as the “true” scores are impossible to be known (see, Angrist et al., 2017 for an investigation on bias in school VA scores).

In the present study, we used low-stakes data from the Luxembourg School Monitoring Programme (LUCET, 2021) to circumvent potential sources of bias that result from pressure due to accountability and explore a more informative use of school VA scores, which we would rather recommend. However, this also leads to a limited possibility of generalizing the findings for countries with a high-stakes use of school VA scores (e.g., the UK, France), as the present findings are for now limited to a country with high student diversity and low-stakes data. As the Luxembourgish school system consists of learning cycles, which usually take 2 years but can be extended to 3 years, the number of students who took part in Grade 1 in 2014 but not in Grade 3 in 2016 was quite high. This could have affected the results because the excluded students had lower achievement, lower SES values, and a higher percentage of students who did not speak the first language of instruction at home than those who met the inclusion criteria. However, students who repeated a grade or who switched schools were also excluded from the VA models in prior research, as this practice is typically applied for accountability purposes. Thus, our data and results largely reflect the reality of how school VA scores are typically estimated. Further research is necessary to investigate methods that have previously been suggested for accounting for co-teaching when estimating VA scores, as these methods sound promising to account for school switching (e.g., the “dosage” method, where a percentage of time spent with each of the teachers is used; Hock & Isenberg, 2017).

In addition, in terms of causality and in contrast to the work by Reardon & Raudenbush (2009), we only took into account one point of causal interpretation based on observed data, whereas other aspects, such as the functional form, were kept constant. Furthermore, the used variables were not centered on the mean (as in, e.g., Reardon & Raudenbush, 2009), which would have made the interpretability of VA scores more straightforward.

As the present study was conducted in a highly diverse and multilingual educational context, the present findings would have to be replicated in a more homogenous setting in order to determine whether the findings are only specific to the diverse setting in Luxembourg or can be generalized to other school systems. Additionally, the difference between time points was 2 years (representing one learning cycle in the Luxembourg school system), whereas in most applications of VA models, the difference between time points is only 1 year, which raises the question of whether these results can be replicated with a longitudinal data set with 1 year between measurement points.

The VA score of a certain school juxtaposes the actual level of achievement attained by students attending a certain school with the achievement level that is expected from students who have the same background characteristics. It is thus implicitly assumed that (in a perfect world) we can control for all relevant factors that affect students’ achievement except for differences in the effectiveness between schools. In this perfect world, the resulting VA score would represent a “pure” measure of a school’s effectiveness. However, as in every data set with human data, there are always unobserved relationships, measurement error, and the reliability of the covariates themselves (e.g., Cook et al., 2009), which may affect VA scores. Hence, the VA scores obtained from the various VA models specified in the present paper represent only an estimate of a school’s effectiveness. Yet, this is also the case in the VA models that are used in practice.

The present study only investigated differences in school VA scores within the same time period. However, previous research has indicated high variability in VA scores over time (e.g., Newton et al., 2010; Sass, 2008). Future research could thus extend the present study by including VA scores over a longer time period to investigate whether there are schools with stable VA scores across time within (or across) models and the extent to which the stability over time is related to the choice of covariates.

## Conclusion

Our study empirically supports several conclusions: First, in line with previous research and common VA practices, prior achievement in the same domain is an important covariate for estimating school VA scores. Second, prior achievement in one domain cannot be replaced by prior achievement from another domain. Third, the amount of explained variance and the consistency of school VA scores may be substantially improved by additionally including covariates of between-school differences in student intake. Differential findings in relation to the outcome domain are (a) prior language achievement and sociodemographic and sociocultural background characteristics in the VA models for math, and (b) sociodemographic and sociocultural background characteristics in the VA models for language. Fourth, even though motivational variables have been highly discussed in models of school learning, they did not incrementally add to the quality of school VA scores. However, this might be different when considering higher grades. Fifth, these findings empirically underscore the idea that school VA scores are sensitive to the selection of covariates because the VA scores may vary considerably within individual schools depending on the covariates included in the school VA model. This in turn can lead to radically different assessments of the effectiveness of the same school, raising further doubts concerning causal interpretations of school VA scores when only prior achievement in the same domain is used as covariate to estimate these scores. We thus recommend using VA models with caution and applying VA scores for informative purposes rather than as a mean to base accountability decisions upon.

## Supplementary Information

Below is the link to the electronic supplementary material.

Supplementary file1 (PDF 198 KB)

Supplementary file2 (PDF 198 KB)

Supplementary file3 (PDF 196 KB)

## Footnotes

1 https://epstan.lu/en/assessed-competences-21/

2 https://epstan.lu/en/assessed-competences-31/

3 https://epstan.lu/en/assessed-competences-21/
