# Supplementary material for: How sensitive are the evaluations of a school’s effectiveness to the selection of covariates in the applied value-added model?
Source: Educ Assess Eval Account. 2022 May 23;35(1):129–64. doi: 10.1007/s11092-022-09386-y (PMC9127485; doi:10.1007/s11092-022-09386-y)
Supplement: Supplementary file 1 — Supplementary file1 (PDF 198 KB) [file 11092_2022_9386_MOESM1_ESM.pdf]

Online Resource 1

Number of Schools Classified at a Certain Benchmark by the Math VA Models in Comparison with the Classification by Model 15 with all Covariates

		Classified by Model 15 (with all covariates) as:		
Model Nr		Needs improvement[a]	Moderately effective[b]	Highly effective[c]
1	Needs improvement	28	10	0
	Moderately effective	10	63	4
	Highly effective	0	4	34
2	Needs improvement	27	11	0
	Moderately effective	11	61	5
	Highly effective	0	5	33
3	Needs improvement	34	4	0
	Moderately effective	4	72	1
	Highly effective	0	1	37
4	Needs improvement	19	19	0
	Moderately effective	17	49	11
	Highly effective	2	9	27
5	Needs improvement	29	9	0
	Moderately effective	9	67	1
	Highly effective	0	1	37
6	Needs improvement	27	11	0
	Moderately effective	11	60	6
	Highly effective	0	6	32
7	Needs improvement	37	1	0
	Moderately effective	1	76	0
	Highly effective	0	0	38
8	Needs improvement	17	21	0
	Moderately effective	18	48	11
	Highly effective	3	8	27
9	Needs improvement	27	11	0
	Moderately effective	11	62	4
	Highly effective	0	4	34
10	Needs improvement	27	11	0
	Moderately effective	11	61	5
	Highly effective	0	5	33
11	Needs improvement	34	4	0
	Moderately effective	4	73	0
	Highly effective	0	0	38
12	Needs improvement	19	19	0
	Moderately effective	16	51	10
	Highly effective	3	7	28
13	Needs improvement	30	8	0
	Moderately effective	8	68	1
	Highly effective	0	1	37
14	Needs improvement	27	11	0
	Moderately effective	11	62	4
	Highly effective	0	4	34

[a]Below the 25th percentile. [b]Between the 25th and the 75th percentiles. [c]Above the 75th percentile.
