# Supplementary material for: How sensitive are the evaluations of a school’s effectiveness to the selection of covariates in the applied value-added model?
Source: Educ Assess Eval Account. 2022 May 23;35(1):129–64. doi: 10.1007/s11092-022-09386-y (PMC9127485; doi:10.1007/s11092-022-09386-y)
Supplement: Supplementary file 2 — Supplementary file2 (PDF 198 KB) [file 11092_2022_9386_MOESM2_ESM.pdf]

Online Resource 2

Number of Schools Classified at a Certain Benchmark by the Language VA Models in Comparison with the Classification by Model 15 with all Covariates

		Classified by Model 15 (with all covariates) as:		
Model Nr		Needs improvement[a]	Moderately effective[b]	Highly effective[c]
1	Needs improvement	28	10	0
	Moderately effective	10	58	9
	Highly effective	0	9	29
2	Needs improvement	34	4	0
	Moderately effective	4	69	4
	Highly effective	0	4	34
3	Needs improvement	34	4	0
	Moderately effective	4	69	4
	Highly effective	0	4	34
4	Needs improvement	27	11	0
	Moderately effective	11	56	10
	Highly effective	0	10	28
5	Needs improvement	29	9	0
	Moderately effective	9	60	8
	Highly effective	0	8	30
6	Needs improvement	37	1	0
	Moderately effective	1	73	3
	Highly effective	0	3	35
7	Needs improvement	38	0	0
	Moderately effective	0	75	2
	Highly effective	0	2	36
8	Needs improvement	26	11	1
	Moderately effective	11	54	12
	Highly effective	1	12	25
9	Needs improvement	29	9	0
	Moderately effective	9	59	9
	Highly effective	0	9	29
10	Needs improvement	34	4	0
	Moderately effective	4	69	4
	Highly effective	0	4	34
11	Needs improvement	34	4	0
	Moderately effective	4	69	4
	Highly effective	0	4	34
12	Needs improvement	28	10	0
	Moderately effective	10	58	9
	Highly effective	0	9	29
13	Needs improvement	29	9	0
	Moderately effective	9	61	7
	Highly effective	0	7	31
14	Needs improvement	37	1	0
	Moderately effective	1	75	1
	Highly effective	0	1	37

[a]Below the 25th percentile. [b]Between the 25th and the 75th percentiles. [c]Above the 75th percentile.
