# Supplementary material for: How sensitive are the evaluations of a school’s effectiveness to the selection of covariates in the applied value-added model?
Source: Educ Assess Eval Account. 2022 May 23;35(1):129–64. doi: 10.1007/s11092-022-09386-y (PMC9127485; doi:10.1007/s11092-022-09386-y)
Supplement: Supplementary file 3 — Supplementary file3 (PDF 196 KB) [file 11092_2022_9386_MOESM3_ESM.pdf]

Online Resource 3

Percentiles Resulting From Different VA Models from the Five Example Schools

Model number	School 1	School 2	School 3	School 4	School 5
School VA Score for Math Achievement in Grade 3					
1	.84	.27	.89	.00	.36
2	.78	.40	.91	.03	.58
3	.88	.16	.89	.00	.45
4	.49	.74	.80	.20	.57
5	.87	.19	.88	.00	.43
6	.80	.36	.91	.01	.66
7	.89	.13	.89	.00	.48
8	.41	.80	.85	.18	.42
9	.83	.28	.89	.00	.33
10	.76	.46	.91	.03	.51
11	.88	.18	.89	.00	.41
12	.49	.78	.84	.11	.47
13	.87	.20	.88	.00	.38
14	.79	.44	.93	.01	.62
15	.89	.14	.91	.00	.47
School VA Score for Language Achievement in Grade 3					
1	.30	.87	.61	.05	.32
2	.38	.83	.72	.01	.47
3	.45	.78	.72	.01	.42
4	.34	.94	.59	.11	.42
5	.55	.81	.66	.03	.32
6	.59	.83	.70	.01	.49
7	.61	.77	.73	.01	.40
8	.24	.95	.69	.13	.35
9	.34	.88	.73	.04	.28
10	.44	.86	.75	.01	.42
11	.47	.82	.75	.01	.40
12	.42	.95	.70	.11	.35
13	.63	.85	.73	.03	.31
14	.63	.85	.76	.01	.42
15	.65	.79	.77	.01	.38
